# Single nucleotide polymorphism-based genome-wide linkage analysis in Japanese atopic dermatitis families

**DOI:** 10.1186/1471-5945-7-5

**Published:** 2007-09-28

**Authors:** Hisako Enomoto, Emiko Noguchi, Shigeruko Iijima, Takenori Takahashi, Kazuhito Hayakawa, Mikako Ito, Toshiyuki Kano, Takeshi Aoki, Yoichi Suzuki, Minori Koga, Mayumi Tamari, Tetsuo Shiohara, Fujio Otsuka, Tadao Arinami

**Affiliations:** 1 Department of Medical Genetics, Graduate School of Comprehensive Human Sciences, University of Tsukuba, Japan; 2 Mito Saiseikai General Hospital, Japan; 3 Department of Dermatology, Graduate School of Comprehensive Human Sciences, University of Tsukuba, Japan; 4 Department of Dermatology, Kyorin University School of Medicine, Tokyo, Japan; 5 Mito Red Cross Hospital, Japan; 6 Ibaraki Prefectural Central Hospital, Japan; 7 Department of Pediatrics, Tsukuba Medical Center Hospital, Japan; 8 Department of Public Health, Chiba University Graduate School of Medicine, Japan; 9 Laboratory of Genetics of Allergic Disease, RIKEN SNP Research Center, Yokohama, Japan

## Abstract

**Background:**

Atopic dermatitis develops as a result of complex interactions between several genetic and environmental factors. To date, 4 genome-wide linkage studies of atopic dermatitis have been performed in Caucasian populations, however, similar studies have not been done in Asian populations. The aim of this study was to identify chromosome regions linked to atopic dermatitis in a Japanese population.

**Methods:**

We used a high-density, single nucleotide polymorphism genotyping assay, the Illumina BeadArray Linkage Mapping Panel (version 4) comprising 5,861 single nucleotide polymorphisms, to perform a genome-wide linkage analysis of 77 Japanese families with 111 affected sib-pairs with atopic dermatitis.

**Results:**

We found suggestive evidence for linkage with 15q21 (LOD = 2.01, NPL = 2.87, *P *= .0012) and weak linkage to 1q24 (LOD = 1.26, NPL = 2.44, *P *= .008).

**Conclusion:**

We report the first genome-wide linkage study of atopic dermatitis in an Asian population, and novel loci on chromosomes 15q21 and 1q24 linked to atopic dermatitis. Identification of novel causative genes for atopic dermatitis will advance our understanding of the pathogenesis of atopic dermatitis.

## Background

Atopic dermatitis (ATOD) is a hereditary, pruritic, inflammatory, chronic skin disease that occurs most commonly in early childhood but can persist or start in adulthood. The prevalence of ATOD has been studied in a wide variety of populations [1], and its frequency ranged from 0.73% to 23% of the study populations. The 12-month prevalence value of symptoms of atopic eczema in Japanese children 6 to 7 years of age was 16.9%, the second highest after Sweden [2]. Living in lower, more tropical latitudes, rural areas, and less industrialized regions correlates with a lower prevalence of ATOD[1]. The etiology of ATOD is not fully understood, but atopy, which is characterized by increased levels of immunoglobulin E (IgE) against common environmental allergens, is considered one of the strongest predisposing factors for ATOD.

ATOD is associated with cutaneous hyperresponsiveness to environmental triggers that are innocuous to healthy individuals [3]. In the acute lesions of ATOD, marked perivascular infiltration of inflammatory cells consisting predominantly of lymphocytes and occasional monocyte-macrophages is frequently observed. In chronic lichenified lesions, there are increased numbers of Langerhans' cells and mast cells in the epidermis, and macrophages dominate the dermal mononuclear cell infiltrate [3]. ATOD and its prevalence are often associated with other clinical atopic manifestations, including asthma, allergic rhinitis, rhinoconjunctivitis, and elevated total and/or allergen-specific serum IgE levels. Nearly 80% of children with ATOD develop allergic rhinitis or asthma, suggesting that allergen sensitization through the skin predisposes subjects to respiratory diseases [3].

ATOD is the result of complex interactions between multiple genetic and environmental factors. Sixty-nine percent of patients with ATOD have one or both of parents affected by ATOD [4], and children have a risk of up to 75% of developing the disease when both parents have ATOD [5]. Twin studies have supported the role of a strong genetic contribution with a concordance rate of 0.72–0.86 in monozygotic twins and 0.21–0.23 in dizygotic twins, indicating high heritability of ATOD [5]. Indeed, the heritability of ATOD was estimated at 0.72 by a Norwegian twin study [6].

To identify susceptibility genes for ATOD, 2 approaches can be applied: candidate gene association study, and genome-wide linkage analysis. Several ATOD candidate genes have been identified. The chromosome 5q region harbors many candidate genes for ATOD, including interleukin (IL) -4 (*IL4*), *IL13*, *IL5*, *IL12B*, and serine protease inhibitor Kazal-type 5 (*SPINK5*) [7]. Other candidate genes include high affinity IgE receptor beta chain gene (*FCER1B*), mast cell chymase gene (*CMA1*), and IL4 receptor alpha chain gene (*IL4RA*)[7]. Recent studies have emphasized the importance of skin barrier function in development of ATOD. Two loss-of-function mutations of the filaggrin gene (*FLG*) were found to be associated with ATOD in 2 independent Caucasian populations [8]. For the second approach, genome-wide linkage analysis, and across the entire genome polymorphic DNA markers positioned at specific intervals along each chromosome are screened for linkage to the disease of interest. Because ATOD is a complex disease with a mode of inheritance that does not follow typical Mendelian laws, parametric linkage analysis, which assumes a genetic model, cannot be applied. Therefore, nonparametric methods, such as the affected sib-pairs method, have been used widely to localize susceptibility genes for common diseases such as ATOD. To date, 4 genome-wide linkage studies have been performed in Caucasian populations, but there have been no large studies in other ethnic groups. Evidence for linkage to ATOD was obtained for several chromosomal regions [9-12]. These linkage studies were performed with highly polymorphic microsatellite markers. The recent development of high-throughput genotyping technologies has allowed us to perform genome-wide linkage studies of single-nucleotide polymorphisms (SNPs). The SNP-based genome-wide linkage study has the potential to be as powerful as traditional microsatellite-based analysis and offers good identification of peak locations for further fine-mapping association analyses [13]. In the present study, we performed genome-wide linkage analysis with 77 Japanese families with at least 2 siblings affected with ATOD. This is the first SNP-based whole-genome linkage study in an Asian population.

## Methods

### Subjects

The probands were patients with ATOD who visited the Dermatology Department of the University Hospital of Tsukuba and dermatology departments of 10 hospitals in Ibaraki, and Dermatology Department of the University Hospital of Kyorin in Tokyo, Japan. A full verbal and written explanation of the study was given to patients and all family members interviewed, and all provided informed consent.

ATOD was diagnosed in subjects according to the criteria of Hanifin and Rajka [14]. Patients all had: pruritus, typical appearance of ATOD, and tendency toward chronic or chronically relapsing dermatitis. The diagnosis of all of the patients that participated in this study was confirmed by a dermatologist.

A total of 77 families (287 individuals and 111 sib-pairs) were included in this study (Table 1). The mean age of the probands and their ATOD-affected siblings was 14 years (range 1–43 years); the mean age of the parents was 45 years (32–78 years). The male: female ratio of the children with ATOD was 1:1. This study was approved by the Ethics Committee of the University of Tsukuba.

**Table 1:** Families' structure included in this study

Parents	2-sibs (No. of sib-pairs)	3-sibs (No. of sib-pairs)	4-sibs (No. of sib-pairs)	total
0	10 (10)	1 (3)	0	
1	15 (15)	0	0	
2	38 (38)	11 (33)	2 (12)	

total	63 (63)	12 (36)	2 (12)	77 (111)

### Genotyping

Genomic DNA was extracted from peripheral blood leukocytes or oral brushed cells using standard protocol. The Illumina SNP-based Linkage Panel IV (Illumina, San Diego, Calif.) was used for genotyping. This panel includes 5,861 SNPs distributed evenly across the genome. The average and median intervals between markers are 503 Kb (0.64 cM) and 301 Kb (0.35 cM), respectively. The Illumina markers were typed with the Illumina BeadStation 500G according to the manufacturer's recommendations. Genotyping of SORCS receptor 3 (SORCS3) was done by GoldenGate assay (Illumina) following the manufacturers' instruction.

### Statistical analysis

Affected sib pair linkage analysis was performed along the entire length of each chromosome with the MERLIN program developed by Abecasis et al [15]. Both the nonparametric linkage (NPL) Z score and nonparametric log of the odds (LOD) score calculated with the Kong and Cox linear model [16] were extracted from the MERLIN runs and used to generate graphic plots of the genome-wide scan results. Because linkage disequilibrium (LD) between closely spaced SNPs can falsely inflate linkage statistics, we used the SNPLINK program [17], which removes LD from the marker sets in an automated fashion.

Empirical P values were calculated for the NPL Z and LOD scores via simulation. MERLIN was used to generate 10,000 replicates of families identical to those in our sample. Markers with similar allele frequencies were also generated under the assumption of no linkage. Linkage analyses were then performed on these unlinked replicates, and peaks of NPL and LOD scores were recorded for each simulation. Simulation studies of our genome scan suggested that LOD > 3.16 would have been expected to occur only once in every 20 genome scans in the absence of linkage and LOD > 1.98 would have been expected to occur once per genome scans. These values correspond to "significant" and "suggestive" thresholds for genomewide significance, as defined by Lander and Kruglyak [18]. Our study had a power of > 99, 0.70, 0.15, and 0.02 to detect a susceptibility locus of λs = 3, 2, 1.5, and 1.25 for ATOD with a genome-wide significance of lod > 3.16. The GeneFinder program [19] was used to obtain 95% confidence intervals for the locations of linked loci. Transmission disequilibrium test (TDT)[20] and pedigree disequilibrium test (PDT) [21]was performed with unphased program. Bonferroni correction was applied for the correction of the multiple testing.

### Tag SNP selection

Tag SNPs were selected with Tagger software [22] implemented in Haploview software [23] with r^2 ^threshold of 0.8 and allele frequencies of 0.1.

## Results

We observed an average minor allele frequency (MAF) of 0.28 and a mean heterozygosity of 0.36 in our Japanese population. These values were identical to those in Asian populations on the datasheet for the Illumina Linkage IV Panel. Among 5,861 SNP genotyped, 151 SNPs were not polymorphic in the Japanese population. The call rate (percentage of successful genotype calls among subjects) was used as a measure of quality. The average call rate was 99.5%, and we excluded 19 SNPs with call rates of less than 90%. The rate of Mendelian inconsistency or impossible recombination identified by the MERLIN program was 0.10% in the families with parents available for genotyping. Because the low heterozygosity of SNPs means that only 37% of genotyping errors will appear as Mendelian inconsistencies[15], the approximate genotyping error rate was estimated to be 0.37%.

**Figure 1:**
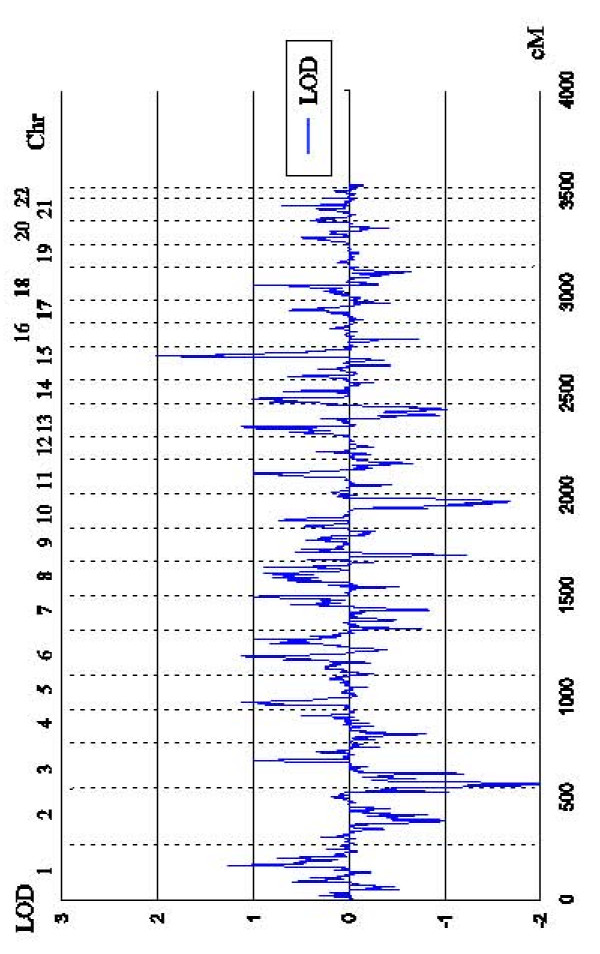
Multipoint nonparametric LOD score of genome-wide scan for ATOD in the Japanese.

Results of the linkage analysis are presented in Figure 1. One region, chromosome 15q21, showed genome-wide suggestive linkage to ATOD (rs2017176, LOD = 2.01, NPL = 2.87, *P *= .0012), with a 95% CI of 49.4 (rs1147129) -76.4 (rs2001597) Mb on the basis of simulation studies. Weak evidence in favor of linkage to 1q24 (rs761076 and rs1933075, LOD = 1.26, NPL = 2.44, *P *= .008) was observed.

Results of TDT and PDT are shown in Table 2. TDT and PDT was family-based test for allelic association. TDT was proposed to test for linkage disequilibrium in family triads, containing two parents and an affected offspring, and PDT is a test for linkage disequilibrium that uses all of the informative data in pedigrees. Table 2 shows 35 SNPs with PDT P values less than 0.01 (uncorrected). However, none of the SNPs reached to a significant association with ATOD after Bonferroni correction. Among 35SNPs showing PDT P value s less than 0.01, 19 SNPs were located in the intergenic region, and most of the SNPs located in intragenic region are intronic SNPs (15 SNPs). We genotyped additional tag SNPs in SORCS3 because two SNPs were associated with ATOD in PDT analysis. Several SNPs were shown to be associated with ATOD (Table 3). One (rs7895087) of the SNPs reached to a significant association with ATOD even after Bonferroni correction, though the number of testing is difficult to determine for this tag SNP association analysis.

**Table 2:** Results of TDT and PDT

rs numbers	Allele	Trans*	Not Trans**	TDT P value (corrected)	PDT P value (corrected)	Gene Name	chr	SNP position
rs717227	T	70	42	0.007818 (1)	0.009023 (1)	CHRM3	1	intron
rs1449504	C	54	21	0.0001059 (0.44)	0.002627 (1)	LRP1B	2	intron
rs1370497	A	35	60	0.009876 (1)	0.006856 (1)	FMNL2	2	intron
rs920891	A	19	36	0.02082 (1)	0.008041 (1)		3	
rs1567058	T	11	30	0.002486 (1)	0.007963 (1)	ARL6	3	Intron
rs1317244	A	48	77	0.009169 (1)	0.007166 (1)	CD200	3	Intron
rs1402276	A	53	30	0.01105 (1)	0.00575 (1)	SCHIP1	3	Intron
rs889319	T	53	80	0.01881 (1)	0.009975 (1)	RAI14	5	Intron
rs40207	T	25	54	0.0009624 (0.99)	0.003361 (1)		5	
rs1552104	T	76	33	0.00002976 (0.15)	0.002873 (1)		5	
rs1498252	A	58	32	0.005775 (1)	0.002604 (1)		6	
rs2894891	A	65	42	0.0256 (1)	0.005798 (1)		6	
rs1158747	T	78	39	0.0002723 (0.78)	0.0008586 (1)	LAMA4	6	Intron
rs169902	A	33	51	0.04865 (1)	0.006104 (1)		7	
rs1202169	A	74	44	0.005487 (1)	0.006487 (1)	ABCB1	7	Intron
rs1419607	A	43	75	0.003033 (1)	0.005659 (1)		7	
rs901592	T	37	70	0.001298 (1)	0.003353 (1)		8	
rs9071	A	36	59	0.01771 (1)	0.007423 (1)	LRRC14	8	3UTR
rs1250288	T	58	36	0.02263 (1)	0.005251 (1)		9	
rs1361800	T	38	15	0.001315 (1)	0.002218 (1)	SORCS3	10	Intron
rs1034178	T	75	42	0.002129 (1)	0.006029 (1)	SORCS3	10	Intron
rs2011505	T	11	28	0.005631 (1)	0.004738 (1)	NCAM1	11	intron
rs3345	T	58	30	0.002604 (1)	0.006276 (1)	HNT	11	intron
rs2034954	A	36	68	0.001559 (1)	0.004317 (1)		12	
rs954108	T	13	28	0.01778 (1)	0.006656 (1)		13	
rs1462256	A	50	79	0.01034 (1)	0.009023 (1)		14	
rs725463	T	65	40	0.01423 (1)	0.00625 (1)		15	
rs1984372	A	58	36	0.02263 (1)	0.008914 (1)		18	
rs1943919	T	37	65	0.005266 (1)	0.008719 (1)		18	
rs2012035	C	30	56	0.004717 (1)	0.009644 (1)		19	
rs542419	T	59	38	0.0323 (1)	0.007665 (1)		19	
rs1477340	T	75	43	0.003033 (1)	0.001527 (1)	NPAS1	19	intron
rs714022	A	50	29	0.01746 (1)	0.001986 (1)	ATXN10	22	intron
rs737822	A	49	26	0.00742 (1)	0.001984 (1)		22	
rs728591	A	42	70	0.007818 (1)	0.004015 (1)		22	

* Number of alleles transmitted to the affected children. ** Number of alleles not transmitted to the affected children. Bonferroni correction was applied for multiple testing

**Table 3:** Results of TDT and PDT by genotypig tag SNPs in SORCS3

rs number	allele	Trans*	Not trans**	TDT P value (corrected)	PDT P value (corrected)
rs768731	C	47	35	0.1843 (0.98)	0.2878 (0.99)
rs790640	C	21	37	0.03446 (0.50)	0.05183 (0.65)
rs790726	G	47	31	0.06905 (0.76)	0.2346 (0.99)
rs791123	G	71	50	0.05563 (0.68)	0.1291 (0.93)
rs971527	G	44	48	0.6766 (1)	0.5258 (1)
rs1472050	T	42	43	0.9136 (1)	0.9178 (1)
rs1490173	G	60	47	0.2083 (0.99)	0.2522 (0.99)
rs1565415	G	50	50	1 (1)	0.9164 (1)
rs1953071	C	52	45	0.4771 (1)	0.3538 (0.99)
rs2491388	A	38	34	0.6373 (1)	0.5675 (1)
rs3011669	A	35	49	0.1257 (0.93)	0.2793 (0.99)
rs4532962	T	51	55	0.6976 (1)	0.6633 (1)
rs7084834	C	19	27	0.237 (0.99)	0.1829 (0.98)
rs7096635	T	53	37	0.09083 (0.85)	0.05368 (0.66)
rs7895087	G	67	29	8.42E-05 (0.0017)	0.0003507 (0.007)
rs9943297	G	44	60	0.1159 (0.91)	0.2017 (0.98)
rs10509784	C	16	39	0.001635 (0.03)	0.007646 (0.14)
rs10509785	C	40	48	0.3934 (1)	0.4579 (1)
rs10884049	G	62	45	0.09957 (0.88)	0.1014 (0.88)
rs11192320	G	41	18	0.002409 (0.047)	0.007646 (0.14)

* Number of alleles transmitted to the affected children. ** Number of alleles not transmitted to the affected children

## Discussion

We performed a genome-wide linkage study using 77 Japanese ATOD-affected families comprising 111 affected sib-pairs (287 individuals) and found 2 candidate linkage regions, on chromosomes 15q21 and 1q24.

This is the first genome-wide linkage study of ATOD in an Asian population, and we did not find much overlap with previously identified linkage regions in Caucasians (Table 4) [9-12]. There are a number of possible reasons for conflicting results in linkage analysis, including differences in ethnic backgrounds, diagnostic criteria, and analytical methods. In the present study, ATOD was diagnosed by dermatologic specialists and followed the criteria of Hanifin and Rajka [14], that was used in previous studies [9-12].

Examining SNPs, instead of microsatellite markers for linkage is unlikely to yield different results because it has been reported that SNP-based genome-wide linkage study has the potential to be as powerful as traditional microsatellite-based analysis and offers good identification of specific locations for further fine-mapping association analysis [13]. We performed a genome-wide linkage study with 5861 SNP markers, although previously performed ATOD genoeme-wide scans have been done with smaller numbers of microsatellite markers. SNPs are distributed more abundantly and uniformly along the human genome than are microsatellite markers are more reliably typed, and require a smaller sample of DNA. Genome-wide linkage mapping of genes with fixed SNP panels, such as our Golden Gate assay, is a cost-effective and time-saving technology [24]. Several recent studies have found that SNP panels provide higher data quality, more accurate genotyping results and higher information content, and they may also have higher power to detect linkage than do traditionally used panels of microsatellite markers [25,26]. Because LD between closely spaced SNPs can falsely inflate linkage statistics, we remove LD from the marker sets in an automated fashion.

The 15q21 linkage region has not been reported previously as a region associated with ATOD. However, linkage of 15q21 to several other inflammatory diseases including osteoarthritis [8] and macular degeneration [27], has been reported. The 15q21 region contains candidate genes for ATOD such as Mothers against decapentaplegic homolog of 3 (SMAD3). SMAD proteins are involved in biologic responses to TGF-beta and related ligands. Smad3-knockout mice show accelerated cutaneous wound healing with complete reepithelialization, and Smad3-deficient keratinocytes show altered patterns of growth and migration [28].

The 1q24 linkage region includes candidate genes such as T-cell receptor zeta chain isoform 2 precursor (*CD3Z*) and chemokine ligand 2 (*XCL2*). CD3Z plays an important role to recognize the coupling antigen to several intracellur signal-transduction pathways [29]. Antigen recognition is one of the most important events in the pathology of ATOD, especially in the memory T cells that encounter their specific antigen, generating an allergen response, which then activates leukocytes leading to production of several cytokines and atopic skin inflammation [3]. Chemokines have fundamental roles in regulation of several types of T cells, development, homeostasis, and function of the immune systems, especially in leukocyte trafficking. During the multistep process of leukocyte trafficking, chemokine ligand-receptor interactions mediate the firm adhesion of leukocytes to the endothelium and initiate transendothelial migration from the blood vessel into perivascular pockets [30]. From perivascular spaces, matrix-bound sustained chemokine gradients direct skin-infiltrating leukocyte subsets to subepidermal or intraepidermal locations. In ATOD regions, that caused by chemokines recruit pathogenic leukocytes to skin in response to mechanical injury such as scratching [31].

Our linkage region on chromosome 1 was located near 1q21, which was previously reported as a linkage region in a British population [10]. It was reported that the skin barrier is impaired in patients with ATOD [32], and recent studies showed that loss-of-function mutations in *FLG *on 1q21 were associated with ATOD in 2 independent populations [8]. FLG is involved in aggregation of the keratin cytoskeleton, which causes collapse of granular cells into flattened anuclear squames. The condensed cytoskeleton is crosslinked by transglutaminases during formation of the cornified cell envelope, the outermost barrier layer of the skin [33], which prevents water loss and impedes the entry of allergens and infectious agents. 1q21, which has been linked to both ATOD and psoriasis [10], houses a cluster of genes known as the epidermal differentiation complex that encode proteins involved in keratinocyte terminal differentiation [34]. Several genes in this region have been reported to be associated with skin diseases such as psoriasis [35]. Because it is possible that the true disease susceptibility gene is located further away from the actual linkage peak, the 1q21 region may include one or more ATOD susceptibility genes for our Japanese population.

**Table 4:** Whole genome linkage studies for ATOD

Year	Authors	Population	Genotyping	No. of markers	No. of families No. of ASP*	Phenotype	Linkage regions
2007	Present study	Japanese	SNPs	5861	77	ATOD	1q24
					111		15q21
2004	Haagerup	Danish	Microsatellite	446	23	ATOD and specific IgE	3q26-3q24
					N/A		4q15-4q14
							18q11-18q12
2002	Bradley	Swedish	Microsatellite	367	109	ATOD	3p24-22
					206	ATOD and specific IgE	18q21
						ATOD and severity score	3q14
							13a14
							15q14
							17q21
2001	Cookson	British	Microsatellite	385	148	ATOD	1q21
					213		17q25
						ATOD and asthma	20p
2000	Lee	European	Microsatellite	380	199	ATOD	3q21
					N/A		

*ASP; affected sib-pairs

Several candidate genes for ATOD were identified by PDT analysis (Table 3). CD200 and its receptor CD200R are both type I membrane glycoproteins that contain two immunoglobulin-like domains. CD200-CD200R interaction has been shown to be important for regulation of the macrophage lineage. In CD200-deficient mice, there were increased numbers of macrophages in the spleen and the mesenteric lymph nodes, and these macrophages show increased activation [36]. In chronic lichenified lesions of ATOD skin, there is an increased number of Langerhans' cells in the epidermis, and macrophages dominate the dermal mononuclear cell infiltrate, and macrophages are important source of cytokines that cause inflammation of the skin [37]. Another candidate is laminin alpha 4 chain (LAMA4). Laminins are a large family of heterotrimeric extracellular matrix glycoproteins in the basement membrane that promote cell adhesion, migration, differentiation, proliferation, and angiogenesis. Lama4-deficient mice showed deterioration of microvessel growth [38], and LAMA4 are located in the basement membrane zone of capillary vessels and in an area adjacent to fibroblast-like cells [39]. SORCS3 is one of the VSP10 domain-containing receptor, that shares the greatest homology with SORCS1. The function of SORCS3 remains unclear, but several SNPs in *SORCS3 *showed association with ATOD by PDT analysis (Tables 2 and 3). Although not in the linkage region, the results of family-based association study suggest that these genes may be associated with the pathogenesis of ATOD.

In conclusion, we performed the first genome-wide linkage study for ATOD in an Asian population, and identified 2 linkage regions, one on 15q21 and one on 1q24. A recent review suggested that there was no substantial overlap between the genetic architecture of ATOD and that of other atopic diseases, such as asthma, but there is a greater degree of similarity between ATOD and psoriasis [7]. Our linkage region on 15q21 overlaps with regions linked to other inflammatory diseases, suggesting that common inflammatory genes may be located in this region. The results of our genome-wide linkage study may lead to identification of novel genes for ATOD, which would improve our understanding of the pathogenesis of ATOD.

## Conclusion

We report the first genome-wide linkage analysis for ATOD in an Asian population and identified novel loci on chromosomes 15q21 and 1q24 linked to ATOD. The results of our genome-wide linkage study may lead to identification of novel genes for ATOD, which would improve our understanding of the pathogenesis of ATOD.

## Competing interests

The author(s) declare that they have no competing interests.

## Authors' contributions

HE carried out molecular genetic study, participated in the study design and coordination and wrote the draft of the manuscript. SI, TT, KH, MI, TK, TA, YS, MK, MT, TS, and FO carried out molecular genetic studies. EN and TA participated in the design of the study and performed the statistical analysis. All authors read and approved the final manuscript.

## Pre-publication history

The pre-publication history for this paper can be accessed here:


